# Tips for success when using social media for online medical education in dermatopathology

**DOI:** 10.1016/j.jdin.2023.07.008

**Published:** 2023-07-28

**Authors:** Casey Schukow, Adrienne Jordan, Antonina Kalmykova, Alice A. Roberts

**Affiliations:** a Department of Pathology, Corewell Health, Royal Oak, Michigan; b Dermatopathology Laboratory of Central States, Dayton, Ohio; c Medical Laboratory CSD Health Care, Ltd, Kyiv, Ukraine; d Department of Dermatology, East Virginia Medical School, Norfolk, Virginia

**Keywords:** Dermatology, dermatopathology, Facebook, medical education, pathology, social media, McKee

We read with great interest the recently published article by Mukosera et al^1^ regarding their collection of online dermatopathology resources for trainees and subsequent recognition of McKee Derm. We would like to take this opportunity to expand the authors’ discussion to highlight tips for success when using social media for online medical education in dermatopathology, as we have learned through McKee Derm and other online platforms.

Social media has been referred to as medical education’s “double-edged sword”^2^ because it allows users to engage in multidirectional communication as they connect with their online communities. Two fields that have recently experienced substantial social media growth are dermatology and pathology, as noted by recent increases in peer-reviewed literature trends.^3^ Although criticisms and ethical concerns of social media for medical education use still exist, Oltulu et al^4^ eloquently outlined common sense and best-practice guidelines for practitioners when using social media tools for generational vocational communication and teaching pedagogy, such as in dermatopathology.

In May 2017, Dr Phillip McKee, a world-renown dermatopathologist now retired at age 75, created McKee Derm (https://www.facebook.com/groups/mckeederm/) to continue providing accessible fellowship-level dermatopathology education to trainees around the world on Facebook while creating a community to network and share difficult cases from sign-out. Thanks to the group’s administration (which includes authors AJ, AK, and AAR) and strict rules of user participation, potential issues with graphic content and possible sharing of patient health information have been minimized in this safe virtual space (see Table I).

**Table I:** McKee Derm rules for safe user participation and engagement from the group’s administrators. Repurposed for this table and letter with permission∗

*No promotions or spam* Give more to this group than you take. Self-promotion, spam, and irrelevant links are not allowed.
*No advertisement other than dermatopathology meetings* Anyone who advertises anything other than meetings relevant to dermatopathology shall be deleted from the group along with the post.
*Be kind and courteous* We are all in this together to create a welcoming environment. Let’s treat everyone with respect. Healthy debates are natural, but kindness is required.
*No hate speech or bullying* Make sure that everyone feels safe. Bullying of any kind is not allowed, and degrading comments about things, such as race, religion, culture, sexual orientation, gender, or identity will not be tolerated.
*Respect everyone’s privacy* Being part of this group requires mutual trust. Authentic, expressive discussions make groups great, but may also be sensitive and private. What is shared in the group should stay in the group.
*Clinical photographs* Clinical photographs must be posted on the original post. No clinical photographs in the comments section.
*Label each case you post* When you post a case please either label it as “for teaching” or “for diagnostic help.”

∗ An original link to these rules can be accessed here: https://www.facebook.com/groups/mckeederm/about.

From our experiences, we would like to offer readers several more tips that may be of value for educators who engage with trainees or other dermatopathologists while on discussion-based educational social media or other online forums:

1. *When posting a case:* first focus on the completeness of clinical information and the appropriateness/quality of microscopic photographs (ie, take new photographs with a higher-definition camera, which can be done on most cellphones). Images that cause concern for violating patient privacy laws (ie, clinical photographs, including faces or tattoos) should be reassessed before posting on public platforms without proper consent.
2. *When commenting on a case:* it is good to start by creating a differential diagnosis and work through logical thought processes (eg, suggestive morphology, positive, and negative stains). Furthermore, do not be afraid to be wrong! It is a part of the learning process and natural. However, it is courteous to indicate when uncertain about a diagnosis or stain and may be appropriate to disclose your career level when so (eg, first-year resident, fellow).
3. *After your initial case post or comment:* follow-up in a timely manner. It is good to indicate a time frame between posts (eg, 3 days later…) and provide initial links/images for reference. This is important for clarification of the final diagnosis and to facilitate discussion, especially when including new stains, molecular findings, or peer-reviewed guidelines/literature.

With common sense and mindfulness for patient privacy information,^5^ we believe that these tips will help dermatopathologists of all ages to continue successfully using social media for medical education.

## Conflicts of interest

Drs Jordan, Kalmykova, and Roberts are administrators of McKee Derm, but they do not receive financial compensation for their positions. Author Schukow has no conflicts of interest to declare.
